# Pitfalls and perspectives in cancer genomes NGS studies: implications for predictive, preventive and personalized medicine (PPPM)

**DOI:** 10.1186/1878-5085-5-S1-A30

**Published:** 2014-02-11

**Authors:** Ugo Rovigatti

**Affiliations:** 1 Pisa University- Medical School, Pisa, Italy

## 

Great increase in Whole Genome Sequencing (**WGS**) and Next Generation Sequencing (here: **NGS-CA**) approaches were witnessed in the past few years, since cataloging or landscaping all possible cancer aberrations appears to be both scientifically relevant and useful for therapeutic strategies. This search has been performed through Medline using **NGS** with appropriate cancer types/subtypes definitions and paying particular attention to the major overviews from groups on both sides of the Atlantic (i.e., Michael Stratton and Bert Vogelstein: [1] and [2]). Although the most utilized instruments-technologies so far are the *454* pyrosequencing apparatuses and the *Illumina* platform, technology is continuously developing, such as the *Ion-Torrent* machines (see below). The scope here is to overview general pitfalls and perspectives in the whole area of **NGS-CA** studies and to suggest probable trends and prudent recommendations.

## Pitfalls

1. Much fewer reports are present today for pediatric tumors and particularly leukemia/lymphoma in comparison to adult cases (ratio 1:10 / 1:20). This is scientifically counterproductive, as major breakthroughs in our understanding of human cancer often originated from pediatric cancer (also next point). No **WGS-NGS** studies have been published so far on neuroblastoma or other pediatric tumors [3].

2. It is apparent that many mutations appeared before the time of malignant transformation in an age/dependent fashion. Although the “mutator phenotype” [1][2] is still discussed, this already suggests that some “drivers” may be irrelevant for malignancy onset. Further discrepancies concern the proportion of oncogenes/TSG and the strategies for targeting the relative mutations (today essentially PKi) [1][2].

3. NGS-CA are biased toward a genetic analysis of malignancies, defined as **CAN-GEN** approach [4]. I previously have [4] and refer here to **CAN-EPI** for alterations at the epigenetic level (scarcely studied by **NGS-CA**), to **CAN-CHROM** for aneuploidy/variation in chromosomes (difficult to study) and to **UP-CAN** for mechanism(s) upstream-responsible for cancer-aberration induction (essentially ignored by **NGS-CA** studies).

4. Strategies dictated by **NGS-CA** studies (particularly, in cases of alterations in EGFR and ALK in lung cancer and BRAF in melanoma – tested in other tumors) universally showed that the eventual remissions are brief/insufficient. Similarly, so called *“Lazarus effects”* were described approximately ten years ago and are resurrected now for justifying more sequencing expeditions.

## Perspectives and Recommendations

1. Further studies should be performed on pediatric cancer, where age is clearly less important/determining factor and where important breakthroughs could be feasible also in the area of **UP-CAN** ([4],[5]).

2. Technology should be followed with great attention. Introduction of the *equivalent of the PC* for sequencing – i.e., Ion Torrent - could lead to desperately - needed new discoveries.

3. Gene-targeting approaches failures, accepted as *“fait accompli”*, are rebutted by stating that multiple agents should be effective. Although big pharma’s do not test combined agents, this approach should be obviously encouraged.

4. As per Pitfalls and especially in view of the great heterogeneity (*intratumoral*, *intrametastatic*, *intermetastatic and between patients *[1][2]) demonstrated in human cancers, additional approaches beside the **CAN-GEN** and namely the **CAN-EPI**, **CAN-CHROM** and particularly **UP-CAN** should be also pursued [4][5] .
